# Complete genome sequence of *Thermovibrio ammonificans* HB-1^T^, a thermophilic, chemolithoautotrophic bacterium isolated from a deep-sea hydrothermal vent

**DOI:** 10.4056/sigs.2856770

**Published:** 2012-09-26

**Authors:** Donato Giovannelli, Jessica Ricci, Ileana Pérez-Rodríguez, Michael Hügler, Charles O’Brien, Ramaydalis Keddis, Ashley Grosche, Lynne Goodwin, David Bruce, Karen W. Davenport, Chris Detter, James Han, Shunsheng Han, Natalia Ivanova, Miriam L. Land, Natalia Mikhailova, Matt Nolan, Sam Pitluck, Roxanne Tapia, Tanja Woyke, Costantino Vetriani

**Affiliations:** 1 Department of Biochemistry and Microbiology, Rutgers University, New Brunswick, NJ, USA; 2 Institute of Marine and Coastal Sciences, Rutgers University, New Brunswick, NJ, USA; 3 Institute for Marine Science - ISMAR, National Research Council of Italy - CNR, Ancona, Italy; 4 Microbiology Department, Water Technology Center, Karlsruhe - Germany; 5 Joint Genome Institute, Walnut Creek, CA, USA; 6 Los Alamos National Laboratory, Bioscience Division, Los Alamos, NM, USA; 7 Oak Ridge National Laboratory, Oak Ridge, TN, USA

**Keywords:** *Aquificae*, *Desulfurobacteriaceae*, thermophilic, anaerobic, chemolithoautotrophic, hydrothermal vent

## Abstract

*Thermovibrio ammonificans* type strain HB-1^T^ is a thermophilic (T_opt_: 75°C), strictly anaerobic, chemolithoautotrophic bacterium that was isolated from an active, high temperature deep-sea hydrothermal vent on the East Pacific Rise. This organism grows on mineral salts medium in the presence of CO_2_/H_2_, using NO_3_^-^ or S^0^ as electron acceptors, which are reduced to ammonium or hydrogen sulfide, respectively. *T. ammonificans* is one of only three species within the genus *Thermovibrio*, a member of the family *Desulfurobacteriaceae*, and it forms a deep branch within the phylum *Aquificae*. Here we report the main features of the genome of *T. ammonificans* strain HB-1^T^ (DSM 15698^T^).

## Introduction

The genus *Thermovibrio* consists of three validly published, named species: *T. ammonificans* strain HB-1^T^ [1], *T. ruber* strain ED11/3LLK ^T^ [2] and *T. guaymasensis* strain SL19^T^ [3]. All three *Thermovibrio* spp. are anaerobic, chemolithoautotrophic bacteria that grow on mineral salts in the presence of carbon dioxide and hydrogen, reducing nitrate or sulfur to ammonium or hydrogen sulfide, respectively. *T. ammonificans* was isolated from an active high-temperature deep-sea hydrothermal vent located on the East Pacific Rise at 9° North, while *T. ruber* was isolated from shallow water hydrothermal vent sediments in Papua New Guinea and *T. guaymasensis* from a deep-sea hydrothermal vent chimney in the Guaymas Basin [1-3]. Anaerobic chemolithoautotrophic bacteria mediate the transfer of energy and carbon from a geothermal source to the higher trophic levels. These anaerobic primary producers, which depend on inorganic chemical species of geothermal origin (*i.e.,* carbon dioxide, hydrogen and sulfur), are completely independent from photosynthetic processes and represent an important component of the deep-sea hydrothermal vent ecosystem. Furthermore, microorganisms such as *T. ammonificans*, which also couple autotrophic carbon dioxide fixation with nitrate respiration, are of particular interest, as they link the carbon and nitrogen cycle, the latter of which has been under-studied at deep-sea hydrothermal vents. Here we present a summary of the features of *T. ammonificans* strain HB-1^T^ and a description of its genome.

## Classification and features

**Figure 1:**
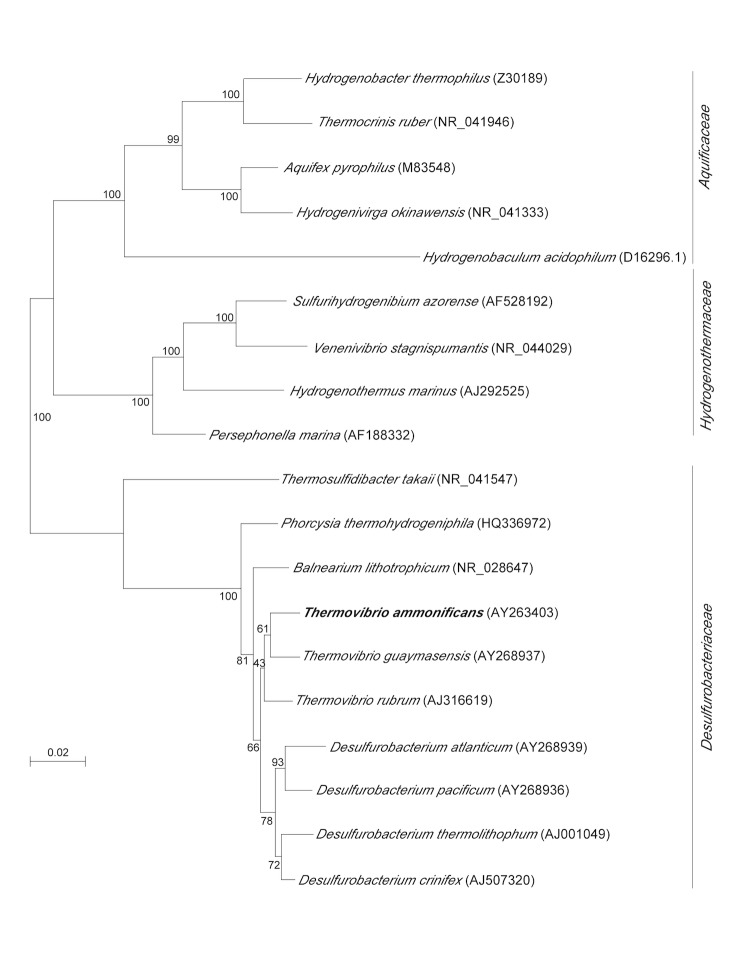
Phylogenetic position of *Thermovibrio ammonificans* HB-1^T^ relative to other type strains within the *Aquificae*. Sequences were aligned automatically using CLUSTAL X and the alignment was manually refined using SEAVIEW [4,5]. The neighbor-joining tree was constructed with Phylo_Win, using the Jukes-Cantor correction [4]. Bootstrap values based on 100 replications. Bar, 0.02 substitutions per nucleotide position.

*Thermovibrio ammonificans* strain HB-1^T^ (=DSM 15698^T^ =JCM 12110^T^) is a member of the phylum *Aquificae*, a group of thermophilic, deeply branching bacteria thought to be among the oldest on Earth. The phylum *Aquificae* consists of a single order, the *Aquificales*, which is composed of three families, *Aquificaceae, Hydrogenothermaceae* and *Desulfurobacteriaceae* (Figure 1). The genus *Thermovibrio* belongs to the family *Desulfurobacteriaceae*, which also includes the genera *Desulfurobacterium*, *Balnearium* and the newly described *Phorcysia* [6-8]. While the genomes of several members of the families *Aquificaceae* and *Hydrogenothermaceae* have been sequenced, the only genome sequences publicly available for the *Desulfurobacteriaceae* are those of *T. ammonificans* and *Desulfurobacterium thermolithotrophum* [9].

**Figure 2:**
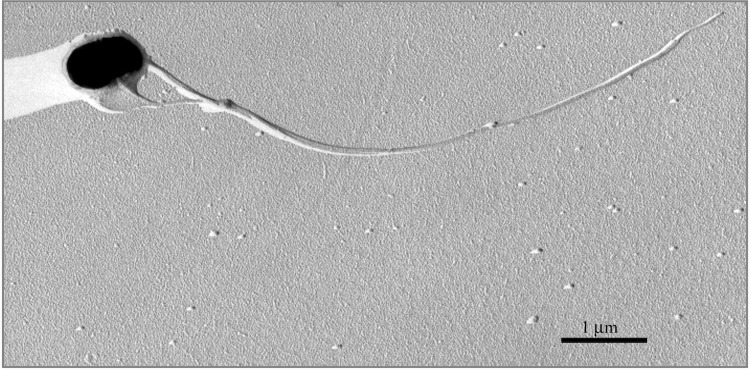
Electron micrograph of a platinum shadowed cell of *Thermovibrio ammonificans* strain HB-1 ^T^ showing multiple flagella. Bar, 1 μm.

Table 1 summarizes the classification and general features of *Thermovibrio ammonificans* HB-1^T^. Cells of *T. ammonificans* are Gram-negative, motile rods of about 1.0 µm in length and 0.6 µm in width (Figure 2). Growth occurs between 60 and 80 °C (optimum at 75 °C), 0.5 and 4.5% (w/v) sodium chloride (optimum at 2%) and pH 5 and 7 (optimum at 5.5). Generation time under optimal conditions is 1.5 h. Growth occurs under chemolithoautotrophic conditions in the presence of hydrogen and carbon dioxide, with nitrate or sulfur as the electron acceptor and with concomitant formation of ammonium or hydrogen sulfide, respectively. Thiosulfate, sulfite and oxygen are not used as electron acceptors. Acetate, formate, lactate and yeast extract inhibits growth. No chemoorganoheterotrophic growth was observed on peptone, tryptone or Casamino acids. The genomic DNA G+C content is 52.1 mol% [1].

**Table 1:** Classification and general features of *Thermovibrio ammonificans* HB-1^T^

**MIGS ID**	**Property**	**Term**	**Evidence code**
	Current classification	Domain *Bacteria*	TAS [10]
		Phylum ‘*Aquificae’*	TAS [11]
		Class *Aquificae*	TAS [12,13]
		Order *Aquificales*	TAS [12,14,15]
		Family *Desulfurobacteriaceae*	TAS [15]
		Genus *Thermovibrio*	TAS [2]
		Species *Thermovibrio ammonificans*	TAS [1]
		Type strain HB-1^T^	
	Gram stain	Negative	TAS [1]
	Cell shape	Short rod	TAS [1]
	Motility	motile	TAS [1]
	Sporulation	non-sporulating	TAS [1]
	Temperature range	60-80	TAS [1]
	Optimum temperature	75	TAS [1]
	Carbon source	CO_2_	TAS [1]
	Energy source	H_2_	TAS [1]
	Terminal electron acceptor	NO_3_^-^, S_0_	TAS [1]
MIGS-6	Habitat	Marine, deep-sea hydrothermal vent	TAS [1]
MIGS-6.3	Salinity	20 g NaCl l^-1^ (range 5 – 45 g NaCl l^-1^)	TAS [1]
MIGS-22	Oxygen	Anaerobe	TAS [1]
MIGS-15	Biotic relationship	free-living	TAS [1]
MIGS-14	Pathogenicity	Not pathogenic	NAS
MIGS-4	Geographic location	East Pacific Rise	TAS [1]
MIGS-5	Sample collection time	April 2000	TAS [1]
MIGS-4.1	Latitude –	9° 50' N	TAS [1]
MIGS-4.2	Longitude	104° 18' W	TAS [1]
MIGS-4.3	Depth	2500 m	TAS [1]
MIGS-4.4	Altitude	not applicable	

Evidence codes - IDA: Inferred from Direct Assay; TAS: Traceable Author Statement (i.e., a direct report exists in the literature); NAS: Non-traceable Author Statement (i.e., not directly observed for the living, isolated sample, but based on a generally accepted property for the species, or anecdotal evidence). These evidence codes are from the Gene Ontology project [16].

### Chemotaxonomy

None of the classical chemotaxonomic features (peptidoglycan structure, cell wall sugars, cellular fatty acid profile, respiratory quinones, or polar lipids) are known for *Thermovibrio ammonificans* strain HB-1^T^.

## Genome sequencing information

### Genome project history

*T. ammonificans* was selected for genome sequencing because of its phylogenetic position within the phylum *Aquificae* and because of its ecological function as a primary producer at deep-sea hydrothermal vents. Sequencing, finishing and annotation were carried out by the US DOE Joint Genome Institute (JGI). Table 2 shows a summary of the project information and its association with MIGS version 2.0 compliance [17].

**Table 2:** Project information

**MIGS ID**	**Property**	**Term**
MIGS-31	Finishing quality	Finished
MIGS-28	Libraries used	454 pyrosequence standard library, 454 Paired End, Illumina
MIGS-29	Sequencing platforms	454 GS FLX Titanium and Illumina GAii
MIGS-31.2	Fold coverage	4,325 ×
MIGS-30	Assemblers	Newbler 2.3, Velvet 0.7.63
MIGS-32	Gene calling method	Prodigal 1.4
	Genome Database release	January 7, 2011
	Genbank ID	NC_014926
	GOLD ID	Gc01577
	Project relevance	Chemosynthetic ecosystems, CO_2_ fixation, Thermophiles
		

### Growth conditions and DNA isolation

*T. ammonificans* was grown in two liters of modified SME medium at 75 °C under a H_2_/CO_2_ gas phase (80:20; 200 kPa) with CO_2_ as the carbon source and nitrate as the electron acceptor [1]. Genomic DNA was isolated from 0.5 - 1 g of pelleted cells using a protocol that included a lysozyme/SDS lysis step, followed by two extractions with phenol:chloroform:isoamyl alcohol (50:49:1) and ethanol precipitation. This procedure yielded about 25 μg of genomic DNA, which was submitted to the DOE JGI for sequencing.

### Genome sequencing and assembly

The genome of *Thermovibrio ammonificans* was sequenced at the DOE JGI [18] using a combination of Illumina [19] and 454 platforms [20]. The following libraries were used: 1) An Illumina GAii shotgun library, which generated 10,255,5615 reads totaling 7,794 Mb; 2) A 454 Titanium standard library, which generated 186,945 reads; and 3) A paired end 454 library with an average insert size of 11.895 +/- 2.973 kb, which generated 115,495 reads totaling 104.7 Mb of 454 data. All general aspects of library construction and sequencing performed at the JGI can be found at the JGI website [21]. The initial draft assembly contained 16 contigs in 2 scaffolds. The 454 Titanium standard data and the 454 paired end data were assembled together with Newbler, version 2.3. The Newbler consensus sequences were computationally shredded into 2 kb overlapping fake reads (shreds). Illumina sequencing data was assembled with VELVET, version 0.7.63 [22], and the consensus sequences were computationally shredded into 1.5 kb overlapping fake reads (shreds). The 454 Newbler consensus shreds, the Illumina VELVET consensus shreds and the read pairs in the 454 paired end library were integrated using parallel phrap, version SPS - 4.24 (High Performance Software, LLC). The software Consed [23] was used in the finishing process. Illumina data were used to correct potential base errors and increase consensus quality using the software Polisher developed at JGI (Alla Lapidus, unpublished). Possible mis-assemblies were corrected using gapResolution (Cliff Han, unpublished), Dupfinisher [24], or sequencing cloned bridging PCR fragments with subcloning. Gaps between contigs were closed by editing in Consed, by PCR and by Bubble PCR (J-F Cheng, unpublished) primer walks. A total of 46 additional reactions and 1 shatter library were necessary to close gaps and to raise the quality of the finished sequence. The total size of the genome is 1,759,526 bp (chromosome and plasmid) and the final assembly is based on 67.7 Mb of 454 draft data, which provide an average 40× coverage of the genome, and 7,284 Mb of Illumina draft data, which provide an average 4,285× coverage of the genome.

### Genome annotation

Genes were identified using Prodigal [25] as part of the Oak Ridge National Laboratory genome annotation pipeline, followed by a round of manual curation using the JGI GenePRIMP pipeline [26]. The predicted CDSs were translated and used to search the National Center for Biotechnology Information (NCBI) nonredundant database, UniProt, TIGRFam, Pfam, PRIAM, KEGG, COG, and InterPro databases. These data sources were combined to assert a product description for each predicted protein. Non-coding genes and miscellaneous features were predicted using tRNAscan-SE [27], RNAMMer [28], Rfam [29], TMHMM [30], and signalP [31].

## Genome properties

**Figure 3:**
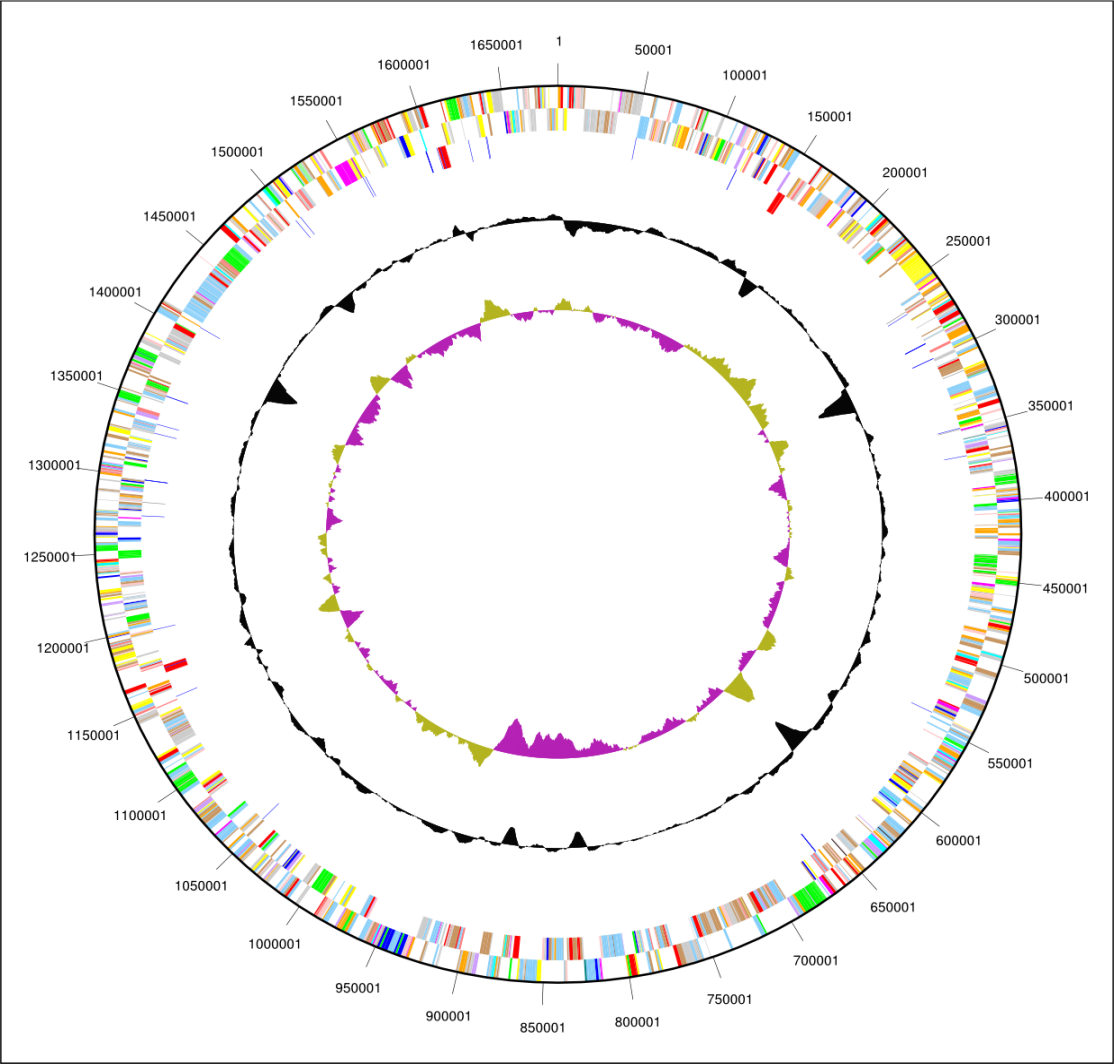
Graphical circular map of the genome. From outside to the center: Genes on forward strand (color by COG categories), Genes on reverse strand (color by COG categories), RNA genes (tRNAs cyan, rRNAs red, other RNAs blue), GC content, GC skew.

The genome includes one circular chromosome and one plasmid, for a total size of 1,759,526 bp (chromosome size: 1,682,965 bp; GC content: 52.13%). Of the 1,888 genes predicted from the genome, 1,831 are protein-coding genes. Of the protein coding genes, 1,279 were assigned to a putative function, with those remaining annotated as hypothetical proteins. The properties and the statistics of the genome are summarized in Figure 3 and Tables 3 and 4.

**Table 3:** Genome statistics

**Attribute**	**Value**	**% of total^a^**
Genome size (bp)	1,759,526	
DNA Coding region (bp)	1,674,589	95.17%
DNA G+C content (bp)	917,237	52.13%
Chromosome (bp)	1,682,965	
Plasmid (bp)	76,561	
Total genes	1888	
RNA genes	57	3.02%
Protein-coding genes	1831	96.98%
Genes in paralog clusters	2	0.11%
Genes assigned to COGs	1419	75.16%
Genes with signal peptides	535	28.34%
Genes with transmembrane helices	369	19.54%
Paralogous groups	1	100%

^a^ The total is based on either the size of the genome in base pairs or the total number of protein coding genes in the annotated genome.

**Table 4:** Number of genes associated with the 25 general COG functional categories

**Code**	**Value**	**% age**^a^	**Description**
J	154	8.41	Translation, ribosomal structure and biogenesis
A	-	-	RNA processing and modification
K	70	3.82	Transcription
L	78	4.25	Replication, recombination and repair
B	3	0.16	Chromatin structure and dynamics
D	40	2.18	Cell cycle control, mitosis and meiosis
Y	-	-	Nuclear structure
V	33	1.80	Defense mechanisms
T	71	3.87	Signal transduction mechanisms
M	135	7.37	Cell wall/membrane biogenesis
N	70	3.82	Cell motility
Z	-	-	Cytoskeleton
W	-	-	Extracellular structures
U	67	3.66	Intracellular trafficking and secretion
O	92	5.02	Posttranslational modification, protein turnover, chaperones
C	158	8.63	Energy production and conversion
G	77	4.20	Carbohydrate transport and metabolism
E	155	8.46	Amino acid transport and metabolism
F	72	3.93	Nucleotide transport and metabolism
H	120	6.55	Coenzyme transport and metabolism
I	46	2.51	Lipid transport and metabolism
P	102	5.57	Inorganic ion transport and metabolism
Q	38	2.08	Secondary metabolites biosynthesis, transport and catabolism
R	220	12.02	General function prediction only
S	92	5.03	Function unknown
-	412	22.50	Not in COGs

^a^ The total is based on the total number of protein coding genes in the annotated genome.
